# Heat‐Suppressing Projection Two‐Photon Lithography Enables High‐Throughput Sub‐Micrometer Manufacturing of Biopolymer Hydrogels for Tissue Engineering

**DOI:** 10.1002/advs.76938

**Published:** 2026-08-24

**Authors:** Qifeng Guan, Yanzhe Fu, Xiangyu Xu, Naitao Li, Sen Hou, Kai Wang, Yufan Wang, Jiakun Li, Ping Li, Jiebo Li, Lizhen Wang, Yubo Fan

**Affiliations:** ^1^ Medical Engineering & Engineering Medicine Innovation Center Hangzhou International Innovation Institute, Beihang University Hangzhou China; ^2^ Key Laboratory of Biomechanics and Mechanobiology of Ministry of Education Beijing Advanced Innovation Center for Biomedical Engineering, School of Biological and Medical Engineering, and with the School of Engineering Medicine, Beihang University Beijing China; ^3^ National Medical Innovation Platform for Industry‐Education Integration in Advanced Medical Devices (Interdiscipline of Medicine and Engineering) Key Laboratory of Innovation and Transformation of Advanced Medical Devices of Ministry of Industry and Information Technology, Beihang University Beijing China

**Keywords:** heat‐suppressing, hydrogels, microparticles, two‐photon polymerization lithography

## Abstract

Native tissues exhibit complex, multiscale hierarchies ranging from centimeter‐scale organization to sub‐micrometer extracellular matrix (ECM) topographies. While two‐photon polymerization (TPP) lithography provides the sub‐micrometer precision essential for mimicking the ECM, traditional point‐scanning TPP is constrained by prohibitively low fabrication efficiency. Although projection two‐photon lithography (P‐TPP) significantly enhances throughput, its application in biopolymer hydrogels is severely hindered by intensive localized heat accumulation, often resulting in material charring and compromised bioactivity. In this study, we developed a heat‐suppressing P‐TPP platform that overcomes these thermal limitations. By employing a low‐exothermic Type II photoinitiating system, we effectively mitigate thermal damage during high‐power polymerization, enabling the high‐fidelity fabrication of hydrogel micro‐units with sub‐micrometer resolution. This technical advancement improves production throughput by 3–4 orders of magnitude compared to conventional point‐scanning TPP. To bridge the gap between micro‐precision and macro‐scale tissue engineering, we further integrated this platform with extrusion‐based printing, a process termed integrated lithography and extrusion additive production. This multiscale manufacturing approach allows for the assembly of engineered micro‐units into sophisticated macroscopic hydrogel constructs that provide critical structural cues for cell alignment and functional tissue maturation. Our platform offers a scalable paradigm for the fabrication of multiscale hydrogels tailored for advanced biomedical applications.

## Graphical abstract

The iLEAP technique integrates high‐throughput two‐photon lithography with extrusion printing, bridging sub‐micrometer precision and macroscopic scaffold fabrication. By employing a low‐exothermic photoinitiator, it mitigates thermal damage to biopolymers and preserves bioactivity. Engineered topographies direct cell alignment, while the approach enables complex, functional tissue constructs. Collectively, these capabilities position iLEAP as a versatile platform for advancing regenerative medicine toward clinical translation.

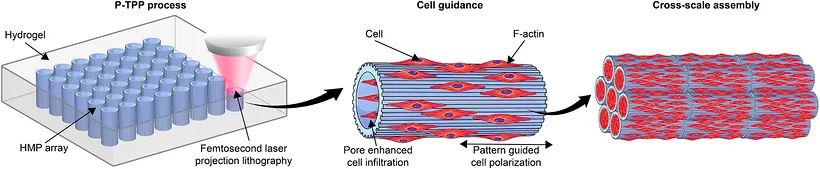

## 1 Introduction

Tissue engineering and bioprinting aim to address critical medical challenges, including organ shortages, wound repair, and drug development, by integrating bioactive materials, functional cells, and advanced fabrication techniques to create biologically functional constructs [1, 2, 3, 4, 5]. Native tissues exhibit a multiscale hierarchy, spanning macroscale tissue organization to cellular alignment and sub‐micrometer extracellular matrix (ECM) features, where the ECM's cross‐scale architecture critically regulates tissue morphogenesis [4, 6, 7]. Hydrogels, though favored for their ECM‐mimetic biocompatibility and nutrient diffusion properties, a long‐standing challenge remains: how to achieve sub‐micrometer structural precision over macroscopic scales with high manufacturing throughput.

Conventional techniques for manufacturing hydrogels suffer from critical drawbacks of limited precision and complexity, as well as incompatibility with precise cell integration [5, 8]. Lithography provides greater design flexibility, yet its resolution and the potential need for complex mask preparation constrain scalability. Digital light processing (DLP) lithography allows rapid microscale patterning, but its optical resolution (∼10 µm) is restricted by light diffraction [9, 10, 11]. Moreover, the inherent nature of hydrogels introduces polymer diffusion and mechanical instability, which further compromise precision—often causing the practical printing resolution to decrease several‐fold relative to the theoretical optical limit [12]. By leveraging a non‐linear two‐photon absorption mechanism, two‐photon polymerization (TPP) lithography effectively bypasses the traditional diffraction limit, achieving a level of precision unattainable with single‐photon systems [13, 14]. This technique utilizes a focused femtosecond laser beam scanned in a serial, point‐by‐point manner to fabricate sub‐micrometer features and intricate 3D geometries within the hydrogel matrix [15]. However, the serial processing nature of the TPP technique renders it inefficient and time‐consuming for fabricating structures beyond the millimeter scale, restricting its use in macroscale applications such as tissue engineering [16]. It is also limited in the dimension of printed structures due to build‐platform or field‐of‐view constraints [17, 18]. To overcome these limitations, projection two‐photon lithography (P‐TPP) has emerged, employing a parallelization strategy that exposes an entire 2D cross‐sectional slice simultaneously; this parallelization strategy dramatically enhances fabrication speed, achieving 3–4 orders of magnitude reduction in production time while still preserving sub‐micrometer resolution [19, 20, 21, 22, 23, 24]. Although P‐TPP utilizes area‐projected femtosecond pulses to maximize throughput, the high energy density required for parallel exposure intensifies optical and chemical proximity effects, which can trigger uncontrolled polymerization [25]. The thermal effects of lasers can cause localized temperature fluctuations of over several hundred K during polymerization [26]. This thermal instability disrupts reaction thresholds and causes material charring, presenting a significant barrier for temperature‐sensitive biopolymer hydrogels.

Here, we report a heat‐suppressing P‐TPP platform designed for the high‐precision and high‐throughput manufacturing of biopolymer hydrogels. By implementing a low‐exothermic Type II photoinitiating system, we effectively mitigate thermal degradation, enabling the fabrication of complex micro‐units (microparticles) with sub‐micrometer resolution. Building upon this advancement, we developed an integrated lithography and extrusion additive production (iLEAP) platform to enable hierarchical tissue fabrication, decoupling high‐resolution manufacturing from macroscopic assembly [27, 28, 29]. Custom‐designed hydrogel microparticles (HMPs) with defined shape, porosity, and surface topography are massively produced via P‐TPP and subsequently assembled through extrusion‐based printing. The HMP‐based bioinks exhibit tailored rheological properties, enabling smooth extrusion, shear‐induced microparticle alignment, and high structural fidelity after printing [29]. By merging two‐photon lithography's precision with scalable extrusion, this hybrid platform overcomes the divide between microscale resolution and centimeter‐scale tissue fabrication (Figure 1). The iLEAP platform thus enables the creation of multi‐scale, cell‐laden hydrogel constructs that recapitulate the hierarchical organization of native tissues from macroscopic architecture to cellular alignment and sub‐micron ECM‐like features. The resulting multiscale hydrogels provide essential structural cues that promote cell alignment and tissue maturation, offering a robust tool for advanced tissue engineering.

**FIGURE 1:**
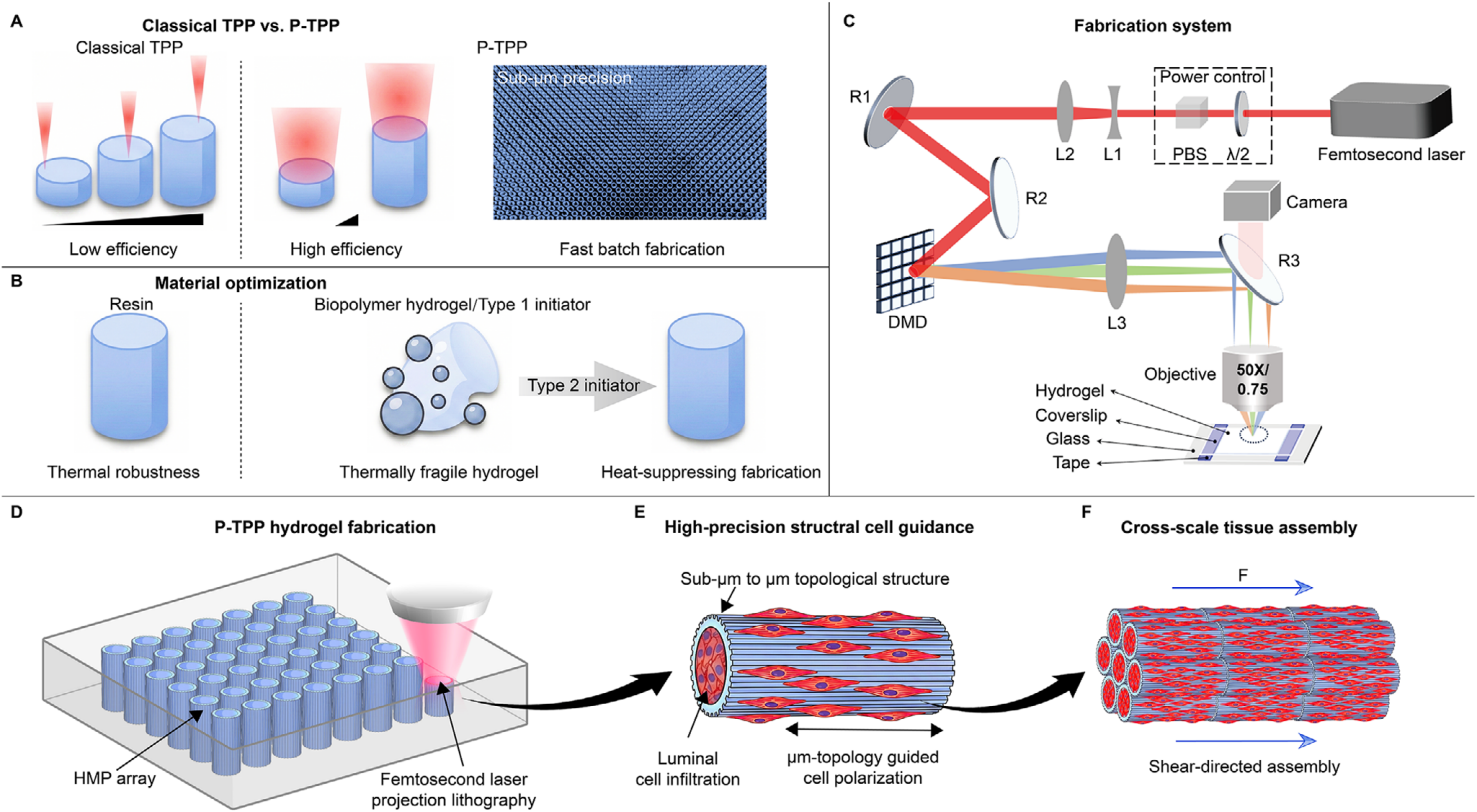
Schematic illustration of the iLEAP process. (A) P‐TPP has much higher efficiency for batch fabrication than the classical TPP process due to a different scanning mode. Scale bar: 100 µm. (B) TPP for natural polymers is more difficult than that of resins due to low cross‐linking speed, resulting in low shape fidelity and heat‐induced bubbles. (C) Schematic diagram of the P‐TPP‐based lithography system for rapidly producing high‐precision HMPs. (D) High‐precision HMPs were rapidly fabricated using projection lithography within a printing ink. The top‐view image of an HMP demonstrates the high fidelity and geometric accuracy achievable with this technique. Scale bar: 10 µm. (E) Surface grooves on the HMPs play a critical role in regulating cell morphology and orientation, while the internal pores facilitate cell infiltration. (F) HMPs can be assembled to enable hierarchical fabrication, integrating microscale cell guidance with macroscopic tissue‐engineering scaffold construction.

## 2 Results

### 2.1 P‐TPP of Hydrogels With Type II Photoinitiating System

The key step of the iLEAP technique involves massively producing HMPs with high precision using P‐TPP lithography. Conventional cleavage‐type photoinitiators (LAP and Irgacure 2959 in Figure S1) fail to achieve optimal hydrogel printing due to premature material charring caused by excessive heat generation prior to reaching targeted polymerization thresholds. To address this, we developed a low‐heat‐generating, Type II photoinitiating system (Eosin Y/triethanolamine) that modulates excited‐state dynamics to achieve uniform polymerization with minimal thermal impact and a broad printing threshold (Figure 2A). Notably, the photoinitiation system has already been widely adopted in biomedical applications and clinical settings due to its excellent biocompatibility [30]. The specific polymerization process of our system is illustrated in Figure 2B. The ink contains the photosensitizer Eosin Y (EY), the electron donor triethanolamine (TEOA), the co‐monomer N‐Vinyl‐2‐pyrrolidone (NVP), and Gelatin Methacryloyl (GelMA). This system remains in an unpolymerized state in the absence of light (Figure S2). When illuminated, the photosensitizer absorbs two photons and transits to an excited state. Subsequently, an electron transfer occurs between the excited state EY and the electron donor, and the electron donor forms a cationic radical. These cationic radicals promote the formation of radicals and cross‐linking of GelMA and NVP to form a hydrogel.

**FIGURE 2:**
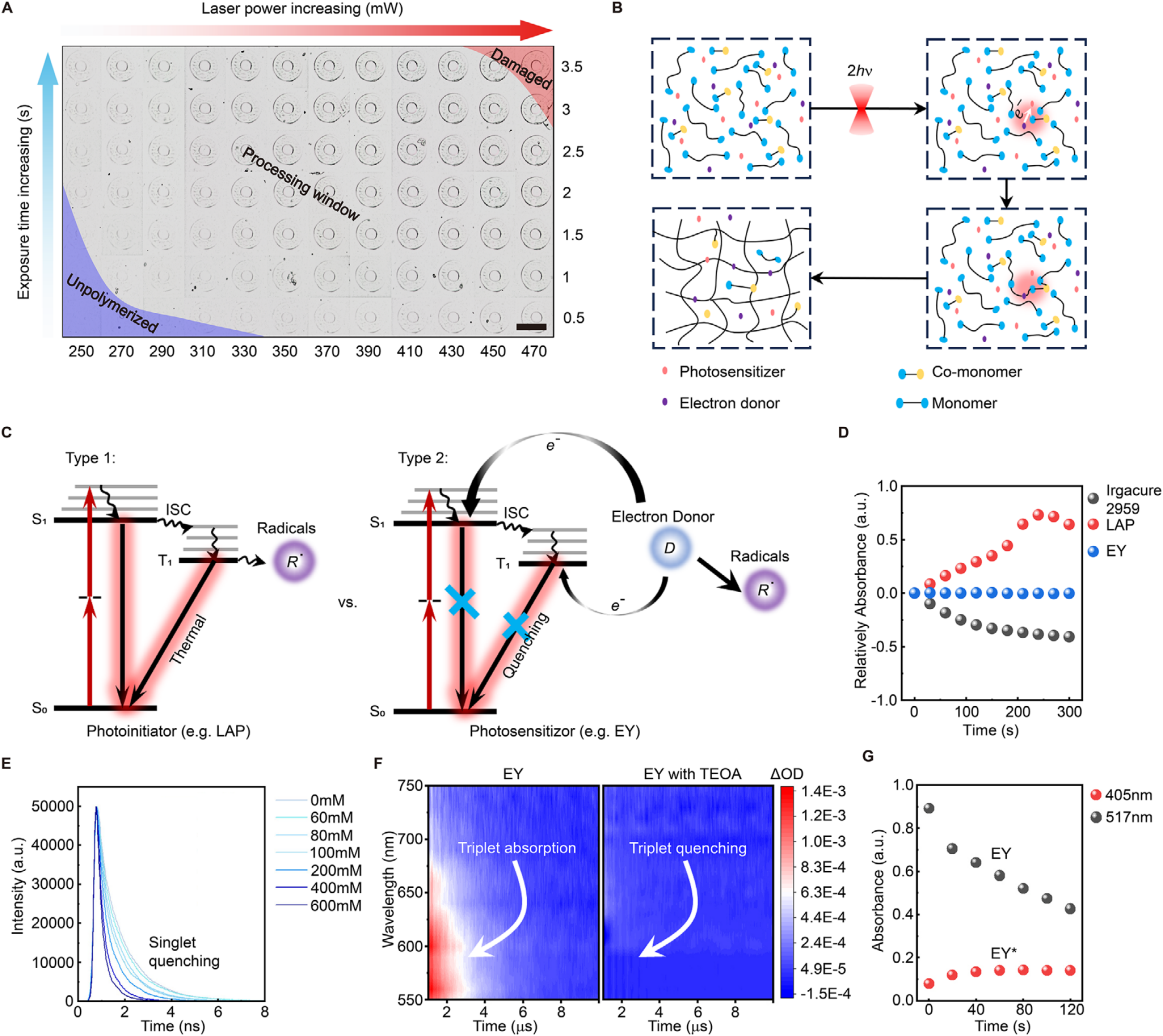
Polymerization mechanism in Type II photoinitiator‐based hydrogels for P‐TPP. (A) Diagram illustrating the direct‐writing window of EY‐based hydrogel inks. Scale bar: 100 µm. (B) Schematic diagram illustrating the bio‐hydrogel polymerization process. (C) Diagrams depicting energy level changes for cleavage‐type and electron‐transfer‐type initiators. (D) Variations in absorption peaks of different initiators over time under light irradiation. LAP: irradiated by 365 nm LED, change in peak intensity at 343 nm; Irgacure 2959: irradiated by deuterium lamp, change in peak intensity at 283 nm; EY: irradiated by 532 nm laser, change in peak intensity at 517 nm. (E) Fluorescence lifetime spectra of mixed solutions containing 10 µm EY and various concentrations of TEOA. (F) Nanosecond flash photolysis results of a solution containing 10 µm EY and a mixed solution containing 10 µm EY and 100 mm TEOA. (G) Temporal intensity changes of absorption peaks at 406 and 517 nm in a mixed solution containing 100 µm EY and 100 mm TEOA under 532 nm laser irradiation.

Unlike cleavage‐type photoinitiators, Type II two‐photon initiating systems offer reduced heat generation (Figure 2C). Cleavage‐type initiators exhibit dual pathways upon S_1_ excitation: non‐radiative decay back to the ground state generates significant heat, while intersystem crossing (ISC) to the T_1_ state enables radical‐producing bond cleavage. This heat accumulation from non‐radiative transitions destabilizes hydrogels (Figure S1B,C). In contrast, within the Type II system, EY molecules excited to S_1_ are predominantly quenched by TEOA. Because the quenching rate resembles the ISC rate, only a small fraction of EY molecules reach the T_1_ state via ISC; these are also rapidly quenched by TEOA, yielding TEOA^+^ radicals. This dual quenching mechanism minimizes non‐radiative transitions and reduces overall system heat.

Spectroscopic analysis reveals distinct behaviors among photoinitiators LAP, Irgacure 2959, and EY under illumination (Figure 2D). UV–vis characterization followed by irradiation at their respective maximum absorption wavelengths (Figure S3) showed that LAP (365 nm LED) and Irgacure 2959 (D_2_ lamp) exhibited decreasing characteristic absorption peak intensities over time, indicating photolytic cleavage, whereas EY (532 nm laser) remains stable. Adding TEOA to EY caused no reaction in the dark (Figure 2E). However, under 450 nm excitation, fluorescence lifetime measurements confirmed TEOA quenches EY's S_1_ state, evidenced by significantly shortened lifetimes as TEOA concentration increases (Figure S5) [31]. Kinetic competition between TEOA quenching and intersystem crossing (ISC) occurs at suboptimal TEOA concentrations; low concentrations allow some EY molecules to reach the triplet (T_1_) state. Nanosecond flash photolysis further demonstrated TEOA's dual quenching: without TEOA, EY showed strong triplet absorption at 580 nm, but upon TEOA addition, this absorption vanished, confirming T_1_ state quenching (Figure 2F and Figure S6). Finally, irradiating the EY/TEOA mixture at 532 nm progressively diminished EY's characteristic 517 nm peak while significantly intensifying a new radical peak by 1.79‐fold at 405 nm (Figure 2G), conclusively demonstrating efficient radical generation via the dual quenching mechanism [32].

### 2.2 HMP Fabrication by P‐TPP Technique

Building on the high photoinitiating efficiency of EY/TEOA for P‐TPP, we next optimized bioink formulation by interrogating how photosensitizer (EY) and polymer (GelMA) concentrations govern cross‐linking kinetics. This parametric screening identified compositions balancing rapid gelation with structural integrity for high‐resolution printing. Using a base 5% (w/v) GelMA solution with fixed concentrations of TEOA (1% v/v) and NVP (5% v/v), we varied EY concentration. Photocuring was performed under 405 nm light (20 mW/cm^2^, 30 s). Compression testing revealed peak hydrogel modulus at 0.4 mm EY (Figure 3A), indicating optimal cross‐linking efficiency, and thus this concentration was selected. We next assessed how GelMA concentration influences printed high‐precision microarchitectures. Under identical printing conditions, 20% (w/v) GelMA produced HMPs with well‐defined geometries. Lower concentrations yielded low‐fidelity HMPs: 10% (w/v) failed to form the target microparticle, while 15% (w/v) resulted in partially deformed particles (Figure 3B). Consequently, 20% (w/v) GelMA was chosen as the optimal ink concentration for further applications. This optimized photoinitiating system also demonstrated broad compatibility across various hydrogel types. It successfully printed protein‐based hydrogels (GelMA 20% w/v, SilMA 10% w/v) and polysaccharide‐based hydrogels (HAA 10% w/v, Dex‐GMA 50% w/v) while maintaining high pattern fidelity (Figure 3C), supporting the system's adaptability to diverse applications (Figure S9).

**FIGURE 3:**
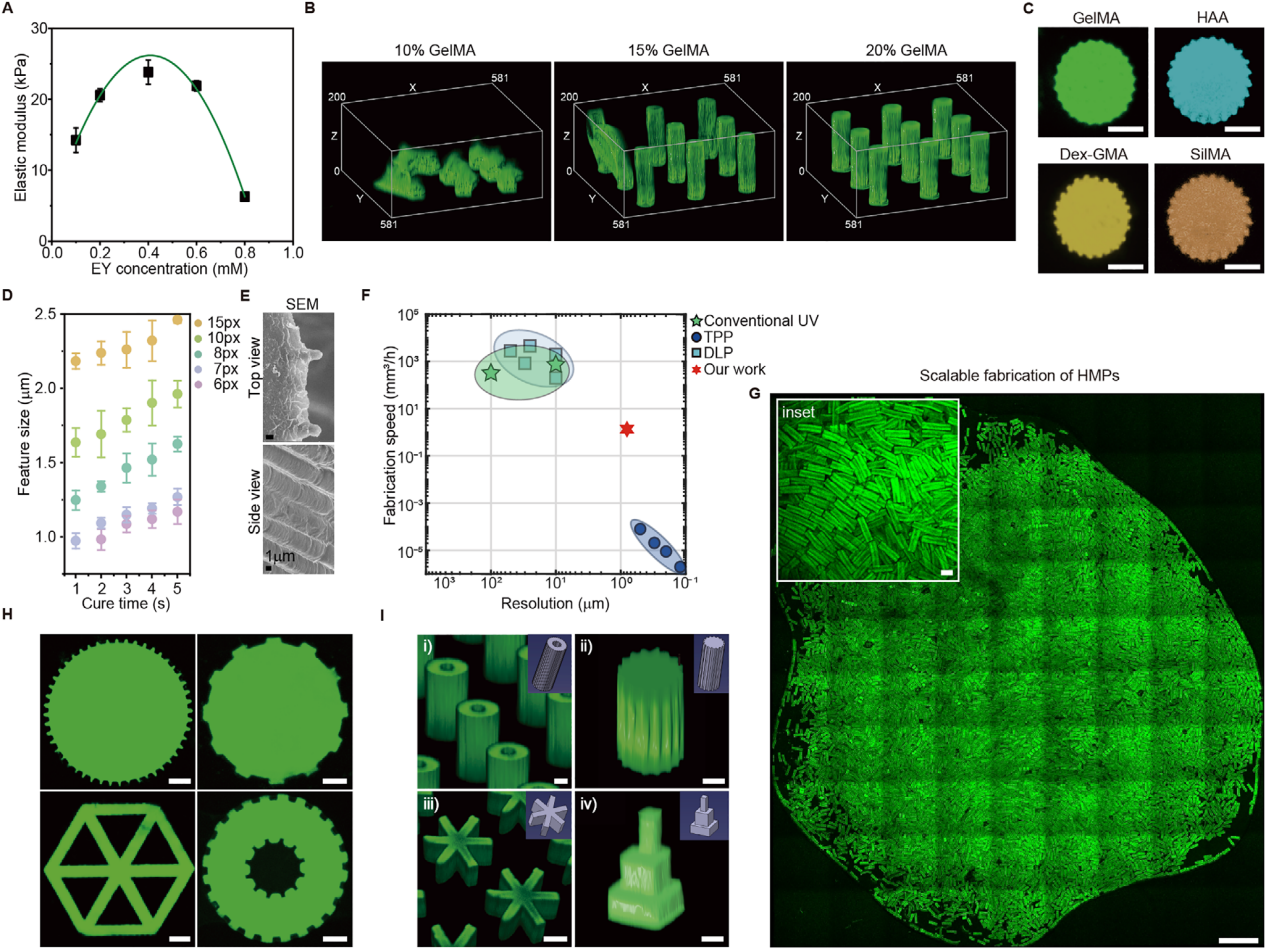
High‐precision, scalable, and material‐adaptive fabrication of HMPs. (A) Compression testing of semi‐cross‐linked printing inks with varying EY concentrations after identical cross‐linking durations (*n* = 3). (B) Printed 3D HMPs with varying GelMA concentrations, demonstrating improved shape fidelity at higher concentrations. (C) Representative top‐view confocal images of HMPs printed using different hydrogels, including GelMA, HAA, Dex‐GMA, and SilMA. Scale bars: 25 µm. (D) Quantitative analysis of the relationship between printed line feature size, pixel width, and exposure time (*n* = 5). (E) SEM images of sub‐micron resolution strips on the surface of HMPs. (F) Comparison of fabrication speed and resolution between the present method and existing bioactive hydrogel manufacturing techniques, including classical two‐photon polymerization for hydrogels (TPP) [34, 35, 36, 37], digital light processing (DLP) [11, 38, 39, 40, 41, 42], and conventional UV Lithography [43, 44]. (G) Z‐stack confocal mosaic image of over 10000 collected tubular HMPs in 1 × PBS, demonstrating that the P‐TPP process is scalable. Scale bars: 1 mm for main image and 100 µm for inset. (H) Printed high‐resolution 2D patterns containing serrations and pores. Scale bars: 10 µm. (I) Fabrication of HMPs with diverse morphologies: (i) tubular HMPs array, (ii) HMPs with surface grooves, (iii) HMPs with sharp edges, (iv) three‐layer structured HMP. Scale bars: 25 µm.

The hydrogel ink's printing accuracy was further quantified through line‐pattern printing experiments. Fabricating line features of varying widths under different exposure durations (laser power: 350 mW) revealed that both lines printed with a 6‐pixel width (2‐s exposure) and 7‐pixel width (1‐s exposure) achieved feature sizes below 1 µm, demonstrating consistent sub‐micrometer resolution (Figure 3D and Figure S10). This high precision was maintained in 3D fabrication, as evidenced by the high‐precision edge structures on HMPs in SEM images (Figure 3E). The performance of this printing system was benchmarked against conventional 3D fabrication technologies, including TPP, DLP, and conventional UV lithography, in terms of processing speed and resolution (Figure 3F). While maintaining sub‐µm resolution, our system achieved approximately 3–4 orders of magnitude increase in fabrication speed compared to TPP (Movies S1 and S2). Although its speed is lower than that of DLP and conventional UV lithography, the achievable resolution far surpasses them. Therefore, this platform offers a unique advantage by balancing high resolution with moderate throughput, outperforming existing methods in overall fabrication efficiency. Importantly, this system enables the large‐scale production of HMPs, demonstrating potential for applications (Figure 3G). This P‐TPP platform achieves a particle fabrication throughput of 815 particles per hour, whereas point‐scanning TPP only produces less than one identical particle per hour. The system also realized exceptional precision in both two‐dimensional (Figure 3H) and three‐dimensional (Figure 3I and Figure S11) patterning, allowing for the creation of complex sub‐micron to microscale hydrogel structures with features such as serrated edges, circular geometries, and sharp corners.

In the second step of the iLEAP process, the high‐precision HMPs are functionalized as bioinks for extrusion‐based printing, which enables the controlled assembly of HMPs into macroscopic, hierarchical constructs. (Figure 4A and Movie S3). During extrusion, the inherent shear forces induced alignment of the HMPs' longitudinal axes along the printing direction, enabling the fabrication of macroscale structures with spatially controlled HMP orientation, which was also proved by simulations in EDEM software (Figure 4B,C) [33]. To systematically optimize this alignment process and printing fidelity, we investigated three key parameters: HMP aspect ratio (Figure 4D,E), HMP concentration (Figure 4F,G), and the extrusion/writing speed ratio (Figure 4H,I). The results demonstrate that effective alignment of HMPs during extrusion printing depends critically on three synergistic parameters. First, HMPs with higher aspect ratios exhibited superior alignment under shear forces, evidenced by reduced angular standard deviations and more consistent orientation within printed lines (Figure 4E). Second, increased HMP concentration enhanced alignment through interparticle interactions that promote directional packing; at low concentration, excessive particle dispersion compromised packing density and disrupted orientation integrity (Figure 4G). Third, the extrusion/writing speed ratio proved pivotal for alignment fidelity. A ratio of ∼20 generated optimal orientation, while lower ratios caused filament breakage due to material starvation. Higher ratios disrupted alignment precision and structural coherence, as excess particles extruded radially outward and accumulated as a clog within the gap between nozzle and substrate, severely disrupting the formation of uniform aligned microstructures (Figure 4I). Furthermore, the structural orientation deteriorates when the writing speed is set to 1 mm/s while the extrusion‐to‐writing speed ratio remains unchanged. In contrast, favorable structural orientation can be maintained at a writing speed of 2 mm/s and above. Considering the biocompatibility requirements for living cell printing, a writing speed of 2 mm/s was selected for all subsequent experiments (Figure S12). In summary, achieving well‐oriented anisotropic scaffolds requires high‐aspect‐ratio HMPs, sufficiently high HMP concentrations, and precise control of the extrusion/writing speed ratio—collectively enabling spatially programmable microarchitectures in macroscale constructs.

**FIGURE 4:**
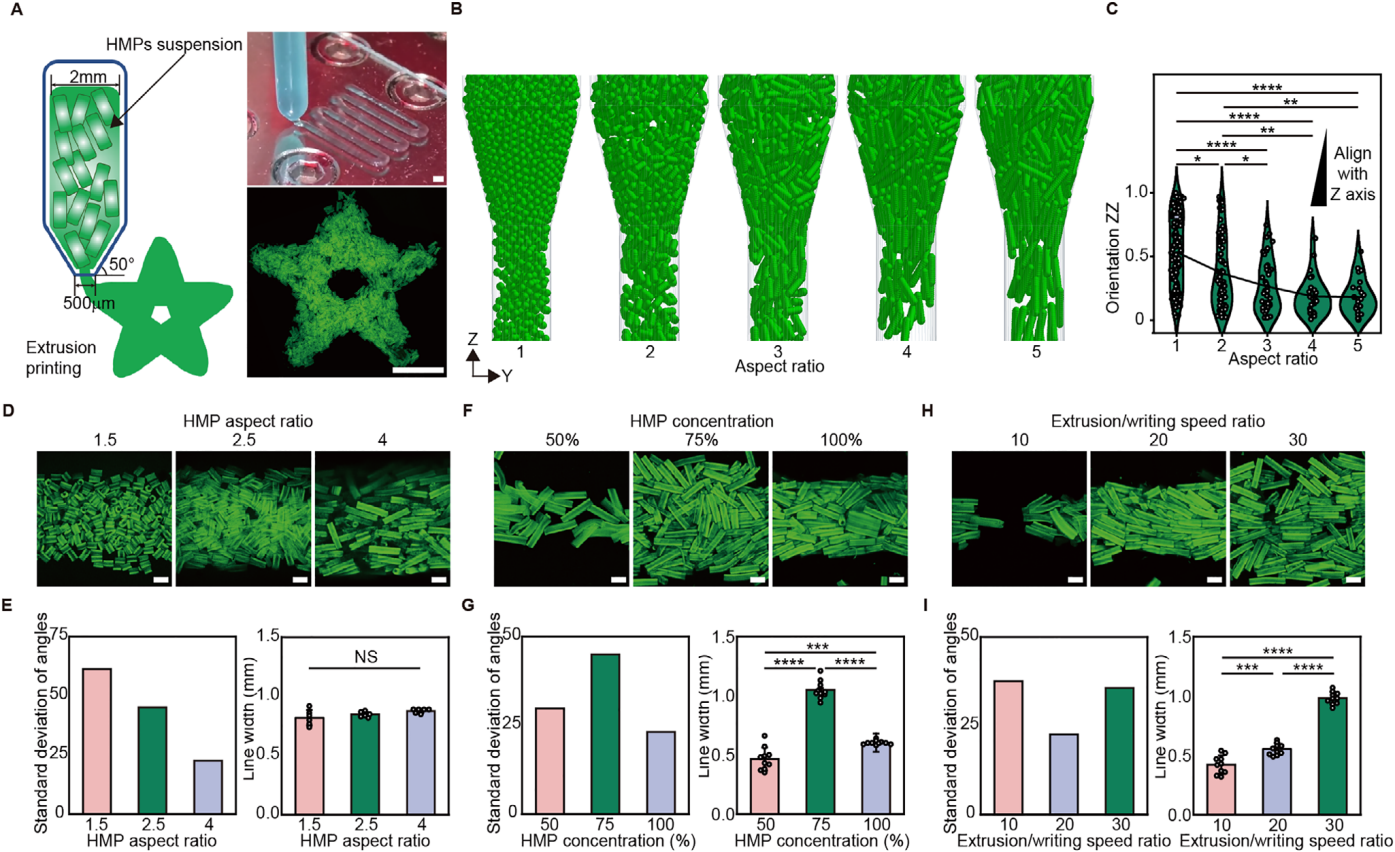
Extrusion printing of HMPs. (A) Extrusion‐based printing of a star‐shaped construct using HMPs as the printing ink. Scale bars: 1 mm. (B) EDEM simulation of how particle aspect ratio influences orientation during extrusion printing. (C) Distribution of particle orientation after extrusion from simulations. (D–I) Parameters affecting HMP orientation and line width for extrusion printing. (D, F, H) Representative maximum intensity projection confocal images of extrusion‐printed lines using HMPs with varying aspect ratios, varying HMP volume fractions, and varying extrusion/writing speed ratios, respectively. Scale bars: 100 µm. (E) Standard deviation of HMP orientation (degree of concentration) and line width for HMPs with different aspect ratios (n > 100 for orientation concentration and *n* = 6 for line width). (G, I) Standard deviation of HMP orientation (degree of concentration) and line width for varying different HMPs volume fractions and varying extrusion/writing speed ratios, respectively (n>100 for orientation concentration and *n* = 10 for line width). NS = not significant, ^*^ = *p* < 0.05, ^**^ = *p* < 0.01, ^***^ = *p* < 0.001, ^****^ = *p* < 0.0001; one‐way ANOVA test.

### 2.3 Customized Porous HMPs Promoting Cell Infiltration and Microtubular Tissue Formation

To emulate the diverse microporous and tubular architectures inherent in native tissues, we aim to fabricate HMPs to replicate these structures. We first designed HMPs with through‐pores of controlled diameters (8, 15, 25, and 50 µm). EDEM simulations were performed to assess how pore size constrains cell invasion. Simulations adopted a simplified linear Hertz–Mindlin contact model, neglecting viscoelastic effects and weak inter‐particle interactions (e.g., van der Waals, electrostatic, and liquid bridging forces). Given the high solid content and stiffness of the HMPs (E > 200 kPa), momentum transfer and geometric constraints dominate, making these simplifications reasonable. Packed HMPs with larger pores permitted substantial cell penetration, whereas those with smaller pores were impenetrable (Figure 5A,B). Differences in pore size also resulted in variations in overall porosity, which in turn affected the ease with which cells could traverse the pore network (Figure 5C). Correspondingly, cells within packed HMPs containing larger pores exhibited more uniform spatial distributions, attributable to their ability to infiltrate the internal pores of individual HMPs (Figure 5D). In contrast, cells in packed HMPs with smaller pores were restricted to migration through interparticle gaps. These HMPs were subsequently fabricated for co‐culture experiments, and live/dead staining confirmed their excellent biocompatibility (Figure S13). After 7 days of culture, cell infiltration occurred exclusively through 25‐µm pores, with approximately 67% of these pores actively infiltrated (Figure S14A,B). Quantitative analysis showed an average nuclear diameter of 15 µm for NIH 3T3 fibroblasts (Figure 3C), indicating that micropores below the size of the nucleus strongly inhibit cell penetration. We next fabricated a series of tubular HMPs with a fixed outer diameter of 90 µm while featuring internal pore diameters of 8, 15, 25, and 50 µm (Figure 5E,F). Consistent with prior observations, no cell infiltration occurred in HMPs with 8‐µm pores. Limited infiltration was observed only at the periphery of 15‐µm pores, whereas robust cellular penetration and spreading occurred within 25‐µm pores. Notably, 50‐µm pores were fully percolated by cells after culture (Figure 5G).

**FIGURE 5:**
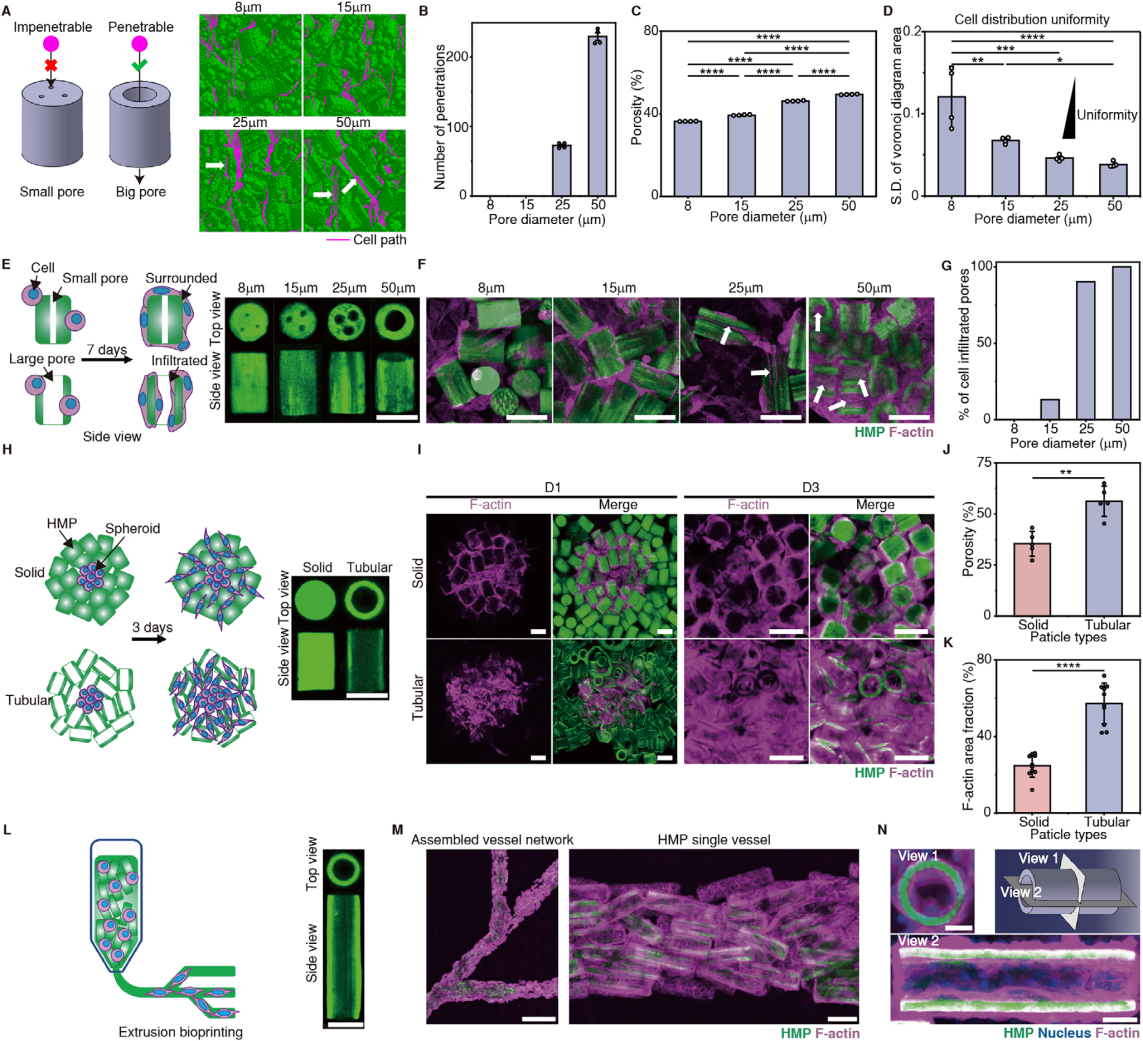
HMP topography and porosity direct cellular infiltration and vasculogenesis in co‐culture. (A) Illustration of pore size‐restricted penetration and representative clipping plane of EDEM simulations of cells passing through packed particles. (B) Number of cell penetrations from multiple simulations (*n* = 4). (C) Porosity of packed particles calculated from multiple simulations (*n* = 4). (D) Cell distribution uniformity calculated from Voronoi diagrams plotted using cell positions in multiple simulations (*n* = 4). (E) Schematic illustration of infiltration assay and confocal images of used HMPs. Scale bar: 100 µm. (F) Representative confocal images of fibroblast infiltration into short HMPs with varying pore size. Scale bars: 100 µm. (G) Quantitative analysis of fibroblast infiltration fractions in short HMPs with different pore sizes. (H) Schematic illustration of spheroid sprouting assay and confocal images of used HMPs. Scale bar: 100 µm. (I) Representative maximum intensity projection confocal images of HUVEC spheroid sprouting in packed solid and tubular short HMPs on day 1 and day 3. Scale bars: 100 µm. (J) Measured porosity of packed solid and tubular short HMPs (*n* = 5). (K) Quantification of F‐actin area fraction within packed solid and tubular short HMPs (*n*  =  10 from *n*  =  2 biological replicates). (L) Schematic diagram of an extrusion‐printed vascular network composed of long tubular HMPs co‐cultured with HUVECs, and confocal images of used HMPs. Scale bar: 100 µm. (M) Maximum intensity projection confocal images of assembled vessel network and a magnified single vessel fabricated using long tubular HMPs and co‐cultured with HUVECs after 5 days of culture. Scale bars: 1 mm for left and 100 µm for right. (N) Two sectional views of a single long tubular HMP from the vascular network structure, with a complete inner endothelial layer. Scale bars: 30 µm. ^*^ = *p* < 0.05, ^**^ = *p* < 0.01, ^***^ = *p* < 0.001, ^****^ = *p* < 0.0001; one‐way ANOVA test for (C, D) and t‐test for (J–K).

Transcending their role as individual particles, HMPs can be assembled into structural scaffolds capable of guiding hierarchical tissue organization. To evaluate how internal pores of HMPs affect cellular network development within these scaffolds, we compared solid (non‐porous) HMPs to tubular HMPs (Figure 5H,I). Both types of HMPs maintained a consistent outer diameter of 90 µm, while tubular HMPs were engineered with an inner through‐pore diameter of 64 µm, yielding a central lumen whose cross‐sectional area precisely matched the annular cross‐section of the tubular structure. Solid HMPs with identical outer dimensions served as architectural controls to isolate the effect of the central lumen. Human umbilical vein endothelial cell (HUVEC) spheroids were co‐cultured with densely packed HMP assemblies to form three‐dimensional hydrogel scaffolds. The tubular HMP arrays provided continuous longitudinal channels for cellular migration and network extension, whereas solid HMPs offered only interstitial spaces between particles for cell infiltration. Porosity analysis revealed that assembled tubular HMPs exhibited an intrinsic porosity of 56%, significantly higher than the 35% observed in solid HMP scaffolds (Figure 5J). After 7 days of culture, confocal imaging and quantitative analysis demonstrated that tubular HMPs supported enhanced cellular outgrowth, with F‐actin coverage reaching 57% compared to only 25% in solid HMP scaffolds (Figure 5K). This disparity is primarily attributed to the pore architecture of tubular HMPs: high intrinsic porosity provides ample free volume for cell migration and proliferation, enabling unconstrained 3D network formation; longitudinal through‐pores establish continuous migration pathways, facilitating rapid cellular infiltration even without hydrogel degradation. In contrast, solid HMPs lack internal pore networks, restricting cells to limited interstitial spaces between particles. Their lower porosity imposes geometric constraints that impede cytoskeletal spreading and collective cell expansion, ultimately suppressing F‐actin polymerization and network maturation.

Beyond their superiority in supporting cell growth, tubular HMPs also exhibit substantial promise for vascular tissue engineering when integrated with extrusion‐based 3D printing. Tubular HMPs with an aspect ratio of 4 were concentrated to a granular bioink and printed using a custom‐designed extrusion printer (Figure 5l and Figure S15). By incorporating HUVECs into the bioink and printing macroscale patterns mimicking vascular geometries, we produced hierarchical structures resembling native vascular networks (Figure 5M). Critically, high‐resolution imaging of individual tubular HMPs revealed the formation of a continuous, vessel‐like endothelial layer within the lumens, clearly visible in both cross‐sectional and longitudinal views (Figure 5N). This demonstrates that the tubular morphology of the HMPs directly guides endothelial cell self‐organization into these vascular‐like microarchitectures. Collectively, these results underscore the feasibility of using tubular HMPs as modular building blocks to engineer complex vascular networks.

### 2.4 Microgrooved HMPs as Topographical Cues for Aligned Tissue Engineering

To mimic the oriented collagen architecture of the ECM and leverage the ultrahigh precision of the P‐TPP technique, we fabricated rod‐shaped HMPs with longitudinal surface microgrooves of controlled widths (Figure 6A). A graded series of microgroove widths was engineered—designated as w2 (2 µm), w4, w10, w20, w30, and a smooth control (w∞)—to systematically evaluate topographical guidance on cell alignment. Primary cells isolated from neonatal Sprague‐Dawley rat brains were co‐cultured with these HMPs for 7 days. Fibronectin (FN) was modified on the surface of HMPs to further enhance cell adhesion and viability (Figure S16). Phalloidin staining revealed that narrow‐groove HMPs (e.g., w2, w4) robustly induced cytoskeletal alignment along the groove direction, evidenced by oriented actin filaments (Figure 6B,C). In contrast, wider grooves (w20, w30) and the smooth control (w∞) exhibited significantly diminished alignment, with cells displaying random orientation. To validate the universality of this topographical effect, NIH 3T3 fibroblasts were cultured on the same HMP series. Consistently, fibroblasts showed enhanced alignment on narrow‐groove HMPs (Figure S17), confirming broad applicability across cell types. These results demonstrate that microgroove width is a critical geometric determinant for cellular orientation. Rod‐shaped HMPs with precisely tuned surface topographies effectively guide anisotropic organization of diverse cell types, offering a biomimetic strategy for engineering aligned tissue constructs.

**FIGURE 6:**
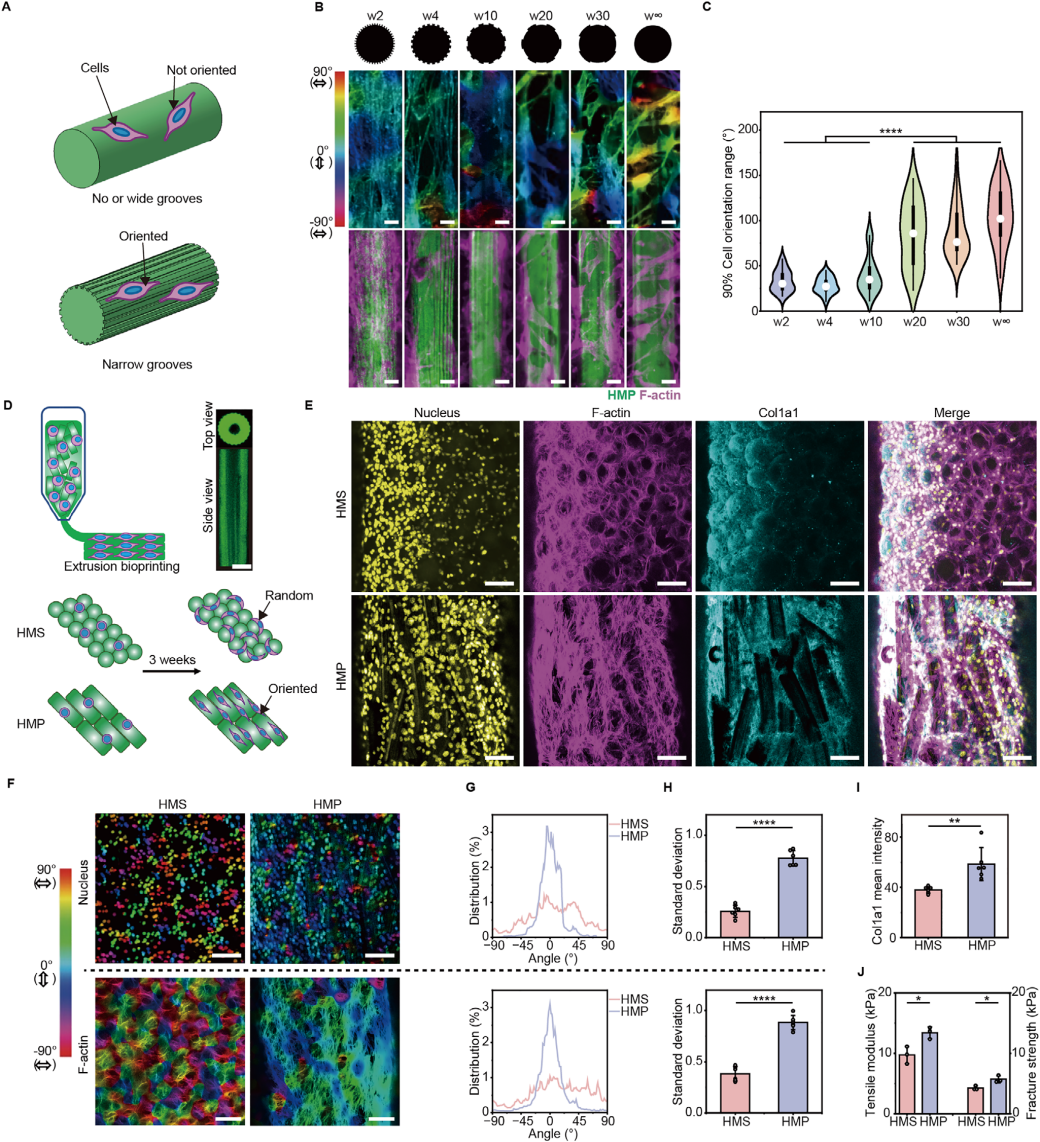
Topographically engineered HMPs for aligned tissue fabrication. (A) Schematic illustration of pattern‐guided cell orientation. (B) Representative confocal images of cortical neurons cultured on HMPs with surface grooves of varying widths. Scale bars: 20 µm. (C) Quantification of the 90% cell orientation distribution range for cortical neurons on HMPs with different groove widths (*n*  =  28–30). (D) Schematic illustration of printed tendon‐like microtissue and confocal images of used HMPs. Scale bar: 50 µm. (E) Representative maximum intensity projection confocal images of NIH 3T3 fibroblasts cultured for 3 weeks on scaffolds assembled from HMSs or grooved HMPs using identical extrusion printing parameters (yellow: nucleus; magenta: F‐actin; cyan: Col1a1). Scale bars: 100 µm. (F) Representative orientation maps of nuclear and F‐actin alignment in HMS‐ versus HMP‐based scaffolds after 3 weeks of culture, with the horizontal direction defined as 0°. Scale bars: 100 µm. (G) Angular distribution of nucleus and F‐actin orientation for both scaffold types. (H) Standard deviation of orientation angles for nucleus and F‐actin, indicating alignment consistency (*n*  =  6). (I) Quantification of Col1a1 fluorescence intensity from single‐slice confocal cross‐sections of the engineered microtissues (*n*  =  6). (J) Tensile modulus and fracture strength measured from uniaxial tensile tests of HMS‐ and HMP‐based scaffolds cultured with NIH 3T3 fibroblasts for 3 weeks (*n*  =  3). ^**^ = *p* < 0.01, ^****^ = *p* < 0.0001; one‐way ANOVA test for (C) and t‐test for (H–J).

Leveraging the natural prevalence of oriented fibroblasts in anisotropic connective tissues (e.g., skin, tendon), we explored whether printed microgrooved HMPs could generate biomimetic anisotropic scaffolds in vitro. NIH 3T3 fibroblasts were combined with the grooved HMPs and co‐printed into defined macroscale architectures using extrusion‐based 3D bioprinting (Figure 6D,E). This process spontaneously aligned the HMPs during deposition, creating scaffolds with inherent anisotropy. For comparison, structurally isotropic hydrogel microspheres (HMSs) underwent identical printing and culture protocols. Quantitative orientation analysis confirmed that the HMPs achieved pronounced directional alignment (evidenced by concentrated angular distribution and lower standard deviation of orientation fraction), while the HMSs maintained isotropic dispersion consistent with their design (Figure S18). Following 21 days of culture, fibroblasts within HMP scaffolds exhibited extensive proliferation and marked cytoskeletal alignment parallel to the printing direction (Figure S19). Strikingly, both F‐actin filaments and nuclei in HMP scaffolds displayed significantly higher orientation levels than those in HMS constructs. In contrast, fibroblasts within isotropic HMS scaffolds demonstrated disordered cytoskeletal networks and nuclei, mirroring the surrounding matrix's lack of anisotropy (Figure 6F–H). Furthermore, immunofluorescence staining revealed substantially greater collagen (Col1a1) deposition within HMP scaffolds compared to HMS controls (Figure 6I). This enhanced structural organization translated to superior mechanical properties. Tensile testing after three weeks showed that HMP scaffolds exhibited significantly higher elastic modulus and fracture strength than HMS constructs (Figure 6J).

### 2.5 Mechanistic Insights Into Topography‐Induced Cell Alignment via Simulations

To elucidate the biophysical mechanisms underlying microtopography‐induced cell alignment, we performed coarse‐grained simulations to examine how cells sense and adapt to micrometer‐scale cues by modulating their contact geometry (Figure 7 and Figure S20). The results indicate that topography directly governs initial adhesion strength and spatial organization. Specifically, convex regions restrict integrin binding to a single groove side, resulting in sparse focal adhesions (FAs), whereas concave regions promote denser and more stable FA formation through multi‐surface contact (Figure 7A). The cellular perception of these topographic cues exhibited a clear size‐dependent threshold, manifesting as four distinct adhesion morphologies (Figure 7B). At the subcellular scale (1 µm), grooves acted as continuous barriers that prevented pseudopod penetration, leading to random orientation. When groove dimensions matched the scale of cellular sensing elements (2–4 µm), cells became partially embedded, inducing strong contact guidance with pronounced polarization and alignment. Conversely, transcellular‐scale grooves (10 µm) caused cells to span across multiple ridges, resulting in fragmented adhesion signals and weakened orientation, while supercellular‐scale grooves (20–30 µm) were perceived as isolated planar surfaces, leading to reduced confinement and random alignment. Quantitative analysis confirmed that at widths of 2 and 4 µm, orientation angles were sharply concentrated parallel to the grooves, accompanied by enhanced cell elongation, whereas other widths produced broad angular distributions and reduced polarization (Figure 7C–E). These findings underscore that effective contact guidance requires topographic feature sizes to match the scale of cellular sensing elements such as filopodia and FAs.

**FIGURE 7:**
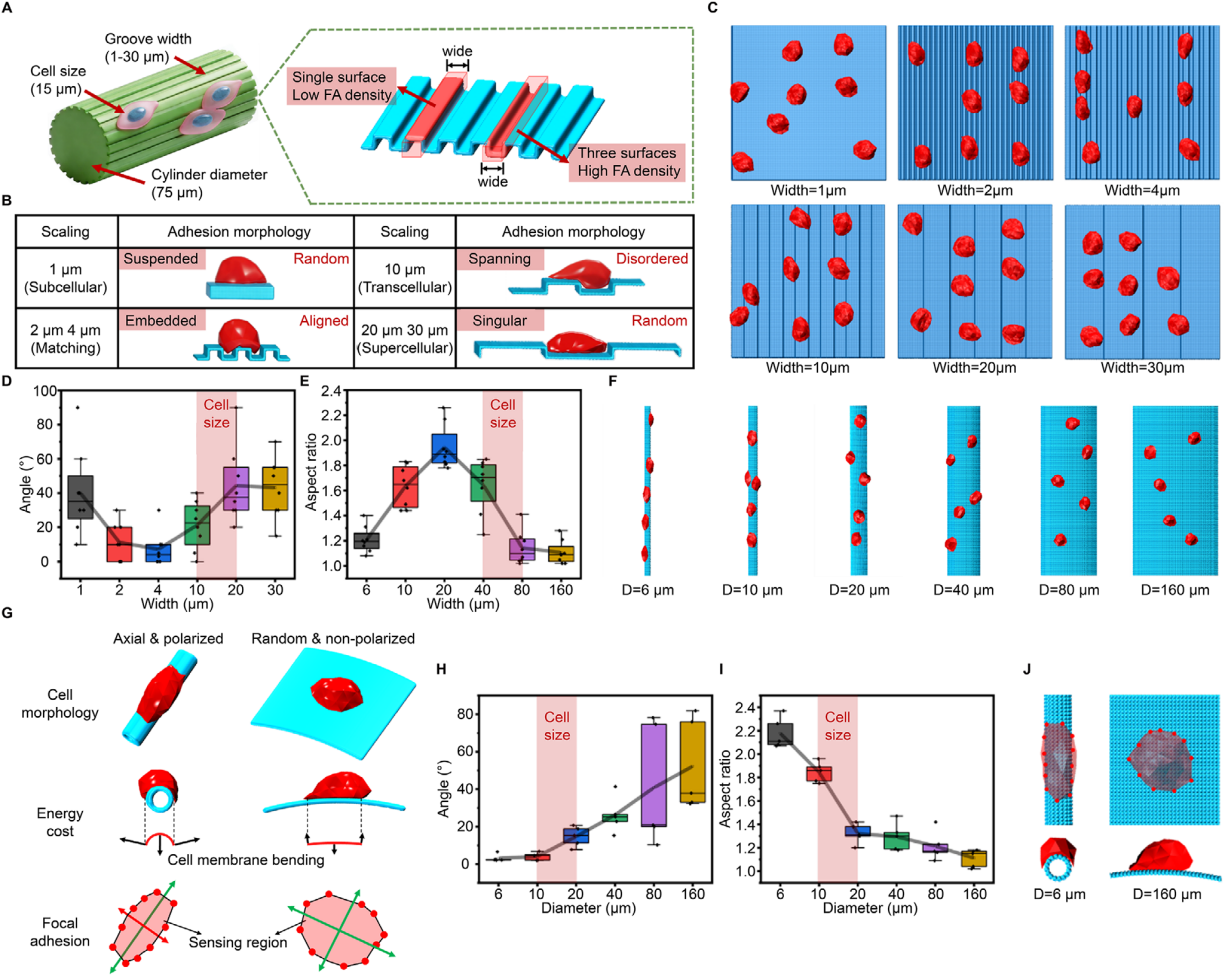
Simulation on the influence of particle morphology on cell adhesion morphology and polarization. (A) Schematic of the model structure. The green part represents a cylindrical microparticle (75 µm in diameter) with parallel surface grooves with widths ranging from 1 to 30 µm. The cell size is approximately 15 µm. The insets on the right illustrate that within the same volume, cells on convex regions contact only one side of the groove, resulting in lower focal adhesion density, whereas cells in concave regions contact three sides, leading to significantly higher focal adhesion density. (B) Four characteristic adhesion morphologies and alignment orientations on a groove with varying width. Suspended morphology (1 µm, random alignment), Embedded morphology (2 and 4 µm, highly aligned along the grooves), Spanning morphology (10 µm, disordered alignment), and Singular morphology (20 and 30 µm, random alignment). (C) Cell alignment and polarization on grooves with different widths. (D) Statistical relationship between groove width and cell alignment angle. (E) Statistical relationship between groove width and cell aspect ratio. (F) Distribution of polarized cell alignment on surfaces with different curvatures (diameter 6–160 µm). (G) On large‐curvature surfaces (diameter 6 µm), cells exhibit distinct polarization along the axial direction, whereas on small‐curvature surfaces (diameter 160 µm), cell orientation is relatively random without obvious polarization. Cells spreading circumferentially on large‐curvature surfaces require overcoming higher membrane bending energy compared to small‐curvature structures. Projections of cell spreading on large‐ and small‐curvature surfaces. In the cell adhesion sensing region, large‐curvature structures show significant curvature gradient anisotropy in the axial and circumferential directions, which in turn determines the anisotropic distribution of focal adhesions. (H) Statistical relationship between cylinder diameter and cell alignment angle. (I) Statistical relationship between cylinder diameter and cell aspect ratio. (J) Top and front views of cell polarization and adhesion on surfaces with diameters of 6 and 160 µm.

To decouple the effects of groove geometry from surface curvature, cells were simulated on cylindrical surfaces with diameters ranging from 6 to 160 µm (Figure 7F,G). A smooth cylinder with a diameter of 75 µm failed to induce alignment, confirming that microgroove structures, rather than curvature alone, drive contact guidance. On high‐curvature cylinders, cells exhibited strong axial alignment and elongated morphologies, whereas on low‐curvature surfaces, alignment became random and polarization diminished (Figure 7H–J). This curvature‐dependent behavior originates from membrane bending energy and curvature‐gradient anisotropy. Since spreading circumferentially on high‐curvature surfaces requires substantial membrane bending energy, axial spreading, which resembles that on a flat surface, is energetically favorable. Consequently, FAs preferentially stabilize in low‐curvature regions, leading to anisotropic adhesion distribution and axial cytoskeletal organization. As the cylinder diameter increases, this anisotropy weakens, resulting in uniform adhesion and a loss of directional guidance.

## 3 Conclusions

The iLEAP technique offers unprecedented hierarchical control by bridging the gap between sub‐micrometer precision and macroscopic tissue fabrication through the integration of high‐throughput, heat‐suppressing projection two‐photon lithography with scalable extrusion printing. By utilizing a low‐exothermic Type II photoinitiating system to prevent thermal damage in biopolymer hydrogels, this platform enables the precise programming of topographical cues—including microgroove widths and surface curvatures—that provide essential structural instructions for guiding cell alignment and functional tissue maturation. This multiscale manufacturing capability provides a versatile strategy for engineering complex functional constructs, from intricate vascular networks to anisotropic connective tissues with enhanced structural maturity and physiological relevance. Furthermore, the precisely tuned biophysical properties of these microparticles hold transformative potential for advanced cell manufacturing, thus establishing a robust foundation for the clinical translation of regenerative medicine and precision biomaterials.

## 4 Experimental Section

### 4.1 Materials

All reagents were purchased from commercial sources without further purification. Porcine gelatin (Type A, ∼300 g Bloom), Irgacure 2959 (98%), and lithium bromide (≥99%) were purchased from Sigma–Aldrich. N‐Vinyl‐2‐pyrrolidone (NVP, 99%) and water‐soluble Eosin Y (ACS) were purchased from Macklin. TEOA (≥98%), dimethyl sulfoxide (DMSO, ≥99.5%), and glycidyl methacrylate (GMA, ≥97%) were purchased from Aladdin. Lithium phenyl (2,4,6‐trimethylbenzoyl) phosphinate (LAP, >98%) and methacrylic anhydride (MA, >97%) were purchased from Tokyo Chemical Industry. Hyaluronic acid (HA, 43 kDa) was purchased from Bloomage Biotech. Dextran (70 kDa), 4‐(4,6‐Dimethoxy‐1,3,5‐triazin‐2‐yl)‐4‐methylmorpholinium chloride (DMTMM, 98%) were purchased from J&K. N‐(2‐Aminoethyl) acrylamide hydrochloride was purchased from Shanghai Yuanye Bio‐Technology Co., Ltd.

Photocurable polymer synthesis: (1) GelMA: Porcine gelatin (10 g) was dissolved in 100 mL 1 × PBS buffer and heated to 50°C. Then, 8 mL of MA was added into the stirred gelatin solution dropwise. After 1 h, 400 mL 1 × PBS buffer was added to end the reaction. The collected solution was dialyzed using a MW 14,000 cut‐off dialysis bag for 4 days and lyophilized for 3 days. Materials were stored in −30°C refrigerator until use. (2) HAA: HA (2 g) was dissolved in 100 mL MES buffer (pH 5.5). Then added 2.91 g DMTMM and 0.79 g N‐(2‐Aminoethyl) acrylamide hydrochloride and adjust the pH to 5.5. After reacting at room temperature, while avoiding light exposure for 48 h, the collected solution was dialyzed using Mw14,000 cut off dialysis bag for 4 days and lyophilized for 3 days. Materials were stored in −30°C refrigerator until use. (3) Dex‐GMA: Dextran (2.54 g) was dissolved in 22.5 mL DMSO. Then, 1.07 mL GMA was added. After a 4‐day reaction at room temperature, the collected solution was dialyzed using Mw1,000 cut off dialysis bag for 4 days and lyophilized for 3 days. Materials were stored in −30°C refrigerator until use. (4) SilMA: 10 g of sliced silkworm cocoon was boiled in 3 L 0.02 m Na_2_CO_3_ solution at 100°C for 30 min. Then they were washed several times with distilled water and dried. They were dissolved in 50 mL of 9.3 m lithium bromide solution at 60°C for 4 h. Then, 3 mL GMA was added and reacted for 3 h at 60°C. The collected solution was dialyzed using Mw 14,000 cut off dialysis bag for 3 days and lyophilized for 3 days. Materials were stored in −30°C refrigerator until use. Proton nuclear magnetic resonance (^1^H NMR, 600 MHz) spectra were acquired using a Jeol JNM‐ECZ400S/L1 spectrometer with D_2_O as the solvent.

### 4.2 Hydrogel Resin Preparation

Coverslips or adhesive tapes were adhered to a slide to control the thickness of printing inks (e.g., single‐layer tape for 25 µm thickness). Printing inks were dropped onto the slide and covered with a coverslip. Each pre‐gel solution contains 0.4 mm EY, 1% (v/v) TEOA, and 5% (v/v) NVP as initiator or copolymer. The concentration for GelMA is 20% (w/v).

### 4.3 Photo‐Physical Characterization

UV–vis absorption spectra were obtained using a violet–visible spectrometer (BIM‐6001A, Brolight). An ultrafast lifetime spectrofluorometer (DeltaFlex, Horiba Jobinyvon IBH Inc) was employed to measure the fluorescence lifetime of EY under excitation by a 450 nm deltadiode in the presence of varying TEOA concentrations. For sub‐microsecond transient absorption (TA) measurements, excitation laser pulses (7 ns, 10 Hz) at 470 nm were generated using an optical parametric oscillator (OPO) driven by a Nd: YAG laser. The visible‐to‐near‐infrared probing light was provided by a white‐light source, which was directed to a monochromator (SP2500i, Princeton Instruments, USA) after interacting with the photoexcited volume. The probe light was detected using a Si‐PIN photodiode (S3071, Hamamatsu Photonics, Japan) attached to the monochromator, and the resulting electrical signal was transmitted to a digital storage oscilloscope (WaveSurfer HDO‐4054, LeCroy Corporation, USA). All samples were placed in quartz cuvettes (ex situ) and analyzed in air.

### 4.4 Rapid HMP Fabrication System Setup

A schematic diagram is shown in Figure S7. Briefly, the light source is a set of femtosecond laser systems (120 fs, 1 kHz, 1.5 W). The power of the laser is adjusted by a set of half‐wave plates (800 nm, MFOPT) and a PBS (MFOPT), and then passed through a beam expander composed of L1 (f = −75 mm, MFOPT) and L2 (f = 50 mm, MFOPT). After reflected by mirrors R1 and R2, it is irradiated onto a DMD (N4100, NSSC, CAS), generating spatial chirp, and irradiated onto the sample surface through a 4‐f system composed of an achromatic lens L3 (AC254‐200‐AB, Thorlabs) and an objective lens. The reflected signal is collected partially by a camera through mirror R3.

### 4.5 HMP Fabrication

The laser power used for fabrication was set to 350 mW. The laser was focused on the bottom of the printing ink by adjusting the position of the Z stage with a single pixel grid picture loaded on the DMD. A series of XY images of HMP cross‐sectional slices was then uploaded to the control unit of the DMD, and the manufacturing of HMP started here. The travel distance along the *Z*‐axis corresponds to the thickness of the printing ink. The exposure was started through the loading of an XY image on the DMD, then the Z stage descended at a speed of 60 µm/s to manufacture HMPs in controlled exposure time. When the target height was achieved, the mirror of the DMD was reset, and the stage moved to the next manufacturing start position, repeat the manufacturing process to achieve array producing. Fabrication of 10,000 HMPs with a height of 250 µm requires 12.27 h using this system, whereas 5.32 h was needed for 10,000 HMPs with a height of 100 µm.

After photocuring, the slide was immersed in 37°C 1 × PBS buffer to remove uncrosslinked pre‐gel solution, with the buffer changed 3 times and 10 min for each time. Then, 50 µL of 0.1%(w/v) LAP dissolved in 1 × PBS buffer was dropped onto HMPs and incubated for 10 min. After that, HMPs were exposed to UV light (20 mW/cm^2^, 405 nm) for 5 min for secondary curing. For fibronectin‐coated HMPs, HMPs on the slide were immersed in 100 µL of 50 µg/mL fibronectin (FN) solution with EDC (5 µg/mL) and NHS (3 µg/mL) cross‐linkers and incubated at 37°C for 1 h. Fibronectin‐coated HMPs were only used for experiments involving primary cortical cells. To label FITC, HMPs on the slide were immersed in 100 µL of 10 µg/mL FITC solution (dissolved in 0.05 m sodium bicarbonate). After each step of coating or labelling, the slide was immersed in 1 × PBS buffer to remove unreacted reagents. To sterilize HMPs, the slide was immersed in 75% ethanol for 24 h. Then washed and immersed in sterile 1 × PBS buffer for storage in 4°C refrigerators.

### 4.6 Feature Size and Confocal Imaging

All confocal images were acquired using a Leica SP8 confocal microscope and LAS X software. To assess printing resolution, lines with varying pixel widths were printed and imaged in bright field under different exposure times. The line widths were measured using FIJI software, with multiple measurements taken across a set of patterns and averaged to determine the characteristic feature size. Z‐stack images of the three‐dimensional structures were acquired with a *Z*‐axis step size of 10 µm, and 3D reconstruction was performed using the Volume Viewer plugin in FIJI software.

### 4.7 Mechanical Tests

A testing machine (Shimadzu, Japan) was used for mechanical tests. For EY concentration optimization, pre‐gel solutions containing 5% (w/v) GelMA, 1% (v/v) TEOA, and 5% (v/v) NVP with different EY concentrations (0.1, 0.2, 0.4, 0.6, and 0.8 mm) were used. 100 µL of pre‐gel solution was added into a cylindrical mold with an inner diameter of 5 mm and cross‐linking using photocuring (20 mW/cm^2^, 405 nm) for 30 s. The cylindrical gel was compressed at 100um/s and force‐displacement curves were recorded. Elastic modulus was calculated using curves between 5% and 10% strain. To evaluate the elastic modulus of printing inks of different hydrogel polymers, each ink was fully cross‐linked under ultraviolet light prior to testing. Compression tests for different hydrogel‐based inks were the same. For tensile tests, the speed was set to 1 mm/min.

### 4.8 Cell Viability Assay

HUVECs were co‐cultured with HMSs and HMPs, respectively. After 1 or 3 days, Calcein AM and Ethidium homodimer‐I (EthD‐I) were added to the culture media to achieve final concentrations of 4 and 2 µm, respectively. Images were acquired after incubation at 37°C for 15 min.

### 4.9 EDEM Simulation

Simulations were performed using EDEM 2020 software to study how particle aspect ratio influences orientation during extrusion printing and to model cell infiltration behavior. First, particles with different aspect ratios were modeled as a linear chain of spheres (70 µm in diameter, spaced 35 µm apart). Particles with aspect ratios of 1, 2, 3, 4, and 5 were composed of 1, 5, 9, 13, and 17 spheres, respectively. The nozzle geometry consists of a conical section (lower diameter = 0.5 mm, upper diameter = 1 mm, height = 2 mm) connected to cylindrical pipes with corresponding sizes at both ends. Particles were randomly generated above the geometry with random orientations and positions, then allowed to fall freely at maximum density. The simulation used a time step of 0.001 s for a total duration of 0.05 s. Afterwards, the orientation of particles in the lower cylinder was quantified using the orientationZZ parameter, where smaller values indicate closer alignment with the *Z*‐axis.

For cell infiltration simulations, particles with pores were randomly generated from a three‐dimensional model of real‐sized particles as templates, with a smoothing value of 2. Cells were modeled as d = 10 µm spheres. The simulation used a time step of 0.01 s for a total duration of 5 s: during the first 2 s, 800 particles fell and accumulated into a 1 mm^3^ cube, reaching a steady state; during the next 3 s, 500 cell‐sized spheres were randomly generated above and allowed to fall freely, and their trajectories were recorded. The volume of packed particles was determined by measuring the upper surface height, and porosity was calculated as the ratio of the total solid particle volume to the occupied space. At 2.3 s, the spatial distribution of cells (spheres) within 0.45–0.5 mm from the bottom surface of the cube was analyzed. Distribution uniformity in the XY plane was evaluated using Voronoi diagrams, where a smaller standard deviation of polygonal areas indicates more uniform cell distribution. Finally, based on cell trajectories, the total number of pore‐penetrating events was counted, where each instance of a cell passing through a pore was recorded as one event.

Viscoelasticity and weak inter‐particle interactions were simplified here. First, the interaction between materials (including HMPs and cells) was described using a simplified linear contact Hertz–Mindlin model and a standard rolling friction model with default settings, without considering its time‐dependent behavior, stress relaxation, or creep. This simplification is justified because during rapid extrusion, the instantaneous contact and packing behavior between particles are far more important than long‐term viscoelastic relaxation. Moreover, when the particle size is small and the extrusion rate is low, the influence of viscoelasticity on the overall flow morphology can be considered a secondary factor. Furthermore, weak inter‐particle interactions are neglected. It is assumed that only frictional and normal/tangential elastic contacts exist between particles, while weak interactions such as van der Waals forces, electrostatic forces, and liquid bridging forces are not taken into account. This is because under extrusion‐based printing conditions, momentum transfer and geometric constraints of stiff HMPs (E > 200 kPa, as shown in Figure S9) dominate the process. Additionally, the bioinks used in the experiments typically have a high solid content, so mechanical contacts between particles are much stronger than surface forces.

### 4.10 Cell Infiltration Assay

First, HMPs with different diameter pores (8, 15, 25, and 50 µm) on each HMP were used. These HMPs were not removed from the slide used during fabrication. Mice NIH 3T3 fibroblasts were directly seeded onto the slide with HMPs on it. Culture medium (DMEM medium containing 10% FBS and 1% penicillin/streptomycin) was changed every 2 days. After culture for 7 days, the slide was immersed in 4% paraformaldehyde to fix the cells. After processed with 0.3% TritonX‐100 for half an hour, the slide was washed with 1 × PBS buffer 3 times before staining with phalloidin (1:200, YP0053S, UElandy) and DAPI in sequence. Images were acquired using a Leica SP8 confocal microscope. The number of cells infiltrated into pores with different diameters was counted. The fraction of infiltrated pores was calculated by dividing the number of infiltrated cells by the total number of pores. The diameter of nuclei was measured using FIJI software. Then, different HMPs with the same diameter pores on each HMP were used analogously. These HMPs were collected and packed with NIH 3T3 cells. Other procedures are the same as described above.

### 4.11 HUVEC Spheroid Sprouting Assay

All the HMPs were 90 µm in diameter. The diameter of the pore in HMPs was 64 µm. HMPs with or without a pore were collected and packed separately. HUVECs spheroids were cultured by adding 1,000 HUVECs into each well of a low‐adhesive 96‐well plate with a U‐shaped bottom and cultured for 3 days. Each spheroid was packed with one kind of HMPs. Collagen I solution (0.5 mg/mL, pH was adjusted to ∼7.0) was added onto packed HMPs to bind HMPs and the spheroid together. After 15 min of incubation, DMEM medium (containing 10% FBS and 1% penicillin/streptomycin) was added for culture. After 1 day and 3 days of culture, cells were fixed and stained as described above. The porosity of packed HMPs was calculated slice‐by‐slice via FIJI software. Briefly, confocal z‐stack images of scaffolds were acquired (*n* = 5). For each packed scaffold, porosity of all slices was calculated, and the average value was set as the porosity of this scaffold. Maximum intensity projection confocal images were used for F‐actin area fraction calculation.

### 4.12 Primary Cell Extraction

The cell extraction method was modified from previous protocols [45, 46]. Briefly, primary cells were extracted from brains of SD rats within 24 h of birth. The front one‐third of the cortex was collected, and meninges were removed completely. The cortex was cut up using sharp scissors and digested using 0.25% pancreatin at 37°C for 15 min. After digestion, the cortex was gently triturated with a Pasteur pipette to release cells. The cell suspensions were filtered by a 40 µm cell strainer, and cells were counted. Temperature for all procedures was 4°C except for digestion.

### 4.13 Oriented Microgroove Guidance of Cell Alignment

For single HMP pattern guidance, rod‐like HMPs with different groove patterns on the side surface were fabricated and coated with FN. The culture dishes were coated with 50 µg/mL FN solution at 37°C for 1 h. These HMPs were collected and put into FN‐coated dishes, with only one pattern in each dish. Then, rat cortical neurons were seeded into dishes at 1 × 10^5^ cells/cm^2^. Neurons were cultured in DMEM medium (containing 20% FBS and 1% penicillin/streptomycin). On day 3, 5 mm cytarabine was added during medium change. After that, medium was changed every 2 days. Cells were fixed and stained on day 7.

### 4.14 Extrusion Printing With Living Cells

The extrusion printing platform was assembled with 4 linear moving stages and designed models to hold everything together. The X and Y stages (step is 2 µm) were placed under the printing nozzle to move the sample for extrusion printing. Z stage (step is 0.74 µm) was placed under the nozzle‐holding arm. The extrusion was achieved through another moving stage (step is 1 µm) connected to the nozzle. The extrusion printing parameters were adjusted by changing the moving speed of these stages. HUVECs and NIH 3T3 fibroblasts were utilized for cell‐laden extrusion printing. HMPs were collected and resuspended in a solution containing 4% (w/v) GelMA, 1% (w/v) HA, and 1 × PBS. The suspension was centrifuged at 13,000 rpm for 60 s, and the centrifugation process was repeated to ensure thorough removal of excess aqueous solution. Subsequently, the cell suspension (1 × 10^7^ cells/mL) was gently mixed with centrifuged HMPs at a volume ratio of 1:20. The resulting mixture was loaded into a Gilson Microman CP100 displacement pipette (used as extrusion nozzle). Extrusion was performed at a printing speed of 100 µm/s and a writing speed of 2,000 µm/s. The gap between nozzle and culture dish was set to 500 µm. The printed structures were then cross‐linked under 405 nm UV light (20 mW/cm^2^) for 60 s to achieve solidification. Following UV exposure, culture medium was added, and the constructs were maintained under standard cell culture conditions. Fibroblast‐seeded samples for tensile tests were 3D printed into strips that are 10 mm long, 1 mm wide, and 1 mm thick.

### 4.15 Orientation Analysis

For HMP orientation after extrusion printing, the angle of the HMP long axis was measured using FIJI software. Confocal images (8‐bit) of cells or materials were analyzed in FIJI software using “OrientationJ Distribution” plugin. The Gaussian window σ was set as 10. Hue, saturation, and brightness were set as orientation, constant, and original‐image, respectively. Other settings were set as default.

### 4.16 Immunofluorescence Staining

Col1a1 rabbit pAb (Abclonal A1352, 1:200) was used for collagen quantification. ABflo 555‐conjugated Goat anti‐Rabbit IgG (Abclonal AS058, 1:200) was used as a secondary antibody. After fixation with paraformaldehyde, the samples were rinsed thoroughly with 1 × PBS multiple times, followed by blocking with 5% goat serum at 37°C for 1 h. The samples were then incubated with the primary antibody at 4°C overnight. After sufficient rinsing, they were subjected to secondary antibody incubation at 37°C.

### 4.17 Coarse‐Grained Model of Cell‐ECM System

We utilized a coarse‐grained (CG) modeling approach to simulate the interaction between cells and ECM, as depicted in Figure S20. Specifically, the CG model represented the membrane of a cell using CG particles, the interaction of which was modeled by bonded or nonbonded interactions. The ECM was also discretized using CG particles that are connected by bonded interactions, which allowed for the formation of a network (Figure S20A). The interaction between cells and ECM fibers is modeled by a Lennard‐Jones potential between particles of the cell and those of fibers within a distance threshold. Three‐dimensional cellular models also need to consider the volume constraints of cells and the bending stiffness of the cell membrane. Figure S20A also illustrates the forces acting on each node during the restoration of cell volume and dihedral angles.

Figure S20B highlights the intracellular forces, intercellular forces, driving forces, and area constraint forces considered in the multi‐cellular model system. The bonded interactions modeling cell membrane tension were referred to as intracellular forces. The non‐bonded interactions between neighboring cells, referred to as intercellular forces, can be disrupted and reformed once broken. The cell structure may be deformed by its active contraction and external forces from neighboring cells within the system. In addition, we considered the cell area as constant; therefore, we applied area constraints on the cell. The driving force for cell migration is generated by the interaction force between the cell and matrix via focal adhesions, called cell traction force [47]. Both experiments and theories indicate that the magnitude of traction force is governed by the so‐called traction‐distance law. Hence, the traction force depends on the cell's size and degree of polarization. For a polarized cell, the traction force is often simplified by a force dipole [48, 49]. The driving force is equal to the net traction force at the critical point of detaching of the cell rear.

Under the influence of these complex mechanical factors, the model is capable of capturing the processes of adhesion, contact, and spreading during cell‐matrix interactions (Figure S20C).

In previous studies, the CG model was extensively utilized to examine collective cell migration on patterned surfaces, wound formation within a single cell layer when subjected to cyclic stretching, and the coupled regulatory effects between cells and the extracellular matrix during tumor invasion [50, 51, 52].

In summary, each node in the model is subjected to multiple forces, including the intercellular force $F_{i}^{inter}$, the intracellular force $F_{i}^{intra}$, the area constraint force $F_{i}^{area}$, the volume constraint force $F_{i}^{volume}$, the active contraction force $F_{i}^{active}$, the driving force of cell motion $F_{i}^{driving}$, the bending resistance force of the cell membrane $F_{i}^{bending}$, and the adhesion force between cell and matrix $F_{i}^{adhesion}$ (as depicted in Figure S20A). The motion of CG particles indexed with *i* (where *i* = 1…N) is governed by the Langevin equation [51, 52].

(1) $$m_{i}\;\frac{d^{2}r_{i}}{dt^{2}}=\;-m_{i}\gamma_{i}\frac{dr_{i}}{dt}+F_{i}^{Resultant}\left(r\right)+\overset{\circ }{\underset{i}{r}}\left(t\right)$$

where the resultant force on each node

(2) $$\begin{array}{cc} & F_{i}^{Resultant}\left(r\right)=F_{i}^{inter}\left(r\right)+F_{i}^{intra}\left(r\right)+F_{i}^{area}\left(r\right)+F_{i}^{volume}\left(r\right)\\ & \;+F_{i}^{active}\left(r\right)+F_{i}^{driving}\left(r\right)+F_{i}^{bending}\left(r\right)+F_{i}^{adhesion}\left(r\right) \end{array}$$

and *m_i_* represents the mass of the particle, while γ_*i*_ is the friction constant. The random force, which is represented by Gaussian white noise to account for the thermal fluctuations, is denoted by $\overset{\circ }{\underset{i}{r}}\left(t\right)$.

$F_{i}^{\mathrm{inter}}$ represents the intercellular force between the CG particles at the cell boundaries, characterizing the cell–cell interaction. The force $F_{i}^{\mathrm{inter}}$ is comprised of the normal and shear components [53, 54].

(3) $$\begin{array}{cc} & f_{i}^{n}=\left\{\begin{array}{cc} \sigma_{c}\left(\frac{r_{ij}^{n}-\delta_{0}}{\delta_{dn}-\delta_{0}}\right)e^{\left(1-\frac{r_{ij}^{n}-\delta_{0}}{\delta_{dn}-\delta_{0}}\right)} & r_{ij}^{n}\leq \delta_{dn}\\ \sigma_{c}\frac{\delta_{fn}-r_{ij}^{n}}{\delta_{fn}-\delta_{dn}} & \delta_{dn}\leq r_{ij}^{n}\leq \delta_{fn}\\ 0 & \delta_{fn}\leq r_{ij}^{n} \end{array};\;\right.\\ & \left.f_{i}^{t}=\left\{\begin{array}{cc} \tau_{c}\frac{r_{ij}^{t}}{\delta_{dt}}e^{\left(\frac{1}{2}-\frac{{\left(r_{ij}^{t}\right)}^{2}}{2{\left(\delta_{dt}\right)}^{2}}\right)} & r_{ij}^{t}\leq \delta_{dt}\\ \tau_{c}\frac{\delta_{ft}-r_{ij}^{t}}{\delta_{ft}-\delta_{dt}} & \delta_{dt}\leq r_{ij}^{t}\leq \delta_{ft}\\ 0 & \delta_{ft}\leq r_{ij}^{t} \end{array}\right.\right. \end{array}$$

where $f_{i}^{n}$ and $f_{i}^{t}$ represent the normal and shear intercellular forces, respectively. Additionally, the maximum values of $f_{i}^{n}$ and $f_{i}^{t}$ are denoted by the parameters σ_c_ and τ_*c*_, respectively. According to previous studies, the parameters σ_c_ and τ_*c*_ were set to be 1100 and 550 pN/µm^2^, respectively [51, 52, 55]. $r_{ij}^{n}$ and $r_{ij}^{t}$ are the normal and tangential distances between particles *i* and *j*, respectively. The equilibrium distance of the intercellular interaction is set as δ_0_ = 0.5 µm. The critical lengths for the maximum values of $f_{i}^{n}$ and $f_{i}^{t}$ are δ_*dn*_ = 1 µm and δ_*dt*_ = 1 µm, respectively. δ_*fn*_ = 2 µm and δ_*ft*_ = 2 µm are the cutoff lengths used to calculate the interaction forces [51, 52].

$F_{i}^{\mathrm{intra}}\left(r\right)$ refers to the intracellular force between particles in the membrane. The intracellular force between the CG particles in the membrane is described as $F_{i}^{\mathrm{intra}}=\left(E_{\mathrm{membrane}}\varepsilon_{ij}\right)\;{\hat{r}}_{ij}$, *E*_membrane_ = 9 × 10^4^ pN/µm is the elasticity of the membrane [51, 52].

The force of the areal constraint is denoted by $F_{i}^{\mathrm{area}}=-\frac{k_{a}\left(A-A_{0}\right)}{A_{0}}\frac{\partial A}{\partial r_{i}}$. The constant of the areal constraint is represented by the parameter *k_a_* = 5 × 10^4^ pN/µm [51, 52, 56]. *A*_0_ refers to the cell area in its original state. The cell area of current state $A=\frac{1}{2}\;\sqrt{{\left(a_{2}b_{3}-a_{3}b_{2}\right)}^{2}+{\left(a_{3}b_{1}-a_{1}b_{3}\right)}^{2}+{\left(a_{1}b_{2}-a_{2}b_{1}\right)}^{2}}$ can be obtained by three particle coordinates *A*(*x*_1_,*y*_1_,*z*_1_), *B*(*x*_2_,*y*_2_,*z*_2_), *C*(*x*_3_,*y*_3_,*z*_3_). Where $\vec{AB}=\left(a_{1},a_{2},a_{3}\right)$, $\vec{AC}=\left(b_{1},b_{2},b_{3}\right)$.

The volume constraint force acting on each node is expressed as $F_{i}^{\mathrm{volume}}=-\frac{k_{v}\left(V-V_{0}\right)}{V_{0}}\frac{\partial V}{\partial r_{i}}$. The volume constraint constant is given by *k_v_* = 2 × 10^4^ pN/µm. Here, *V*_0_ refers to the volume of the cell in its initial state, and *V* denotes the current volume of the cell.

The model explicitly incorporates $F_{i}^{\mathrm{active}}$ to account for the active contraction of the cell. Based on the traction‐distance law and prior experimental findings, the tension force in the cell is approximately proportional to the distance from the cell edge to the cell center [57]. According to earlier studies, the tension force for a polarized cell can be modeled as a force dipole [48, 49]. In this model, the active contraction force is described by $F_{i}^{\mathrm{active}}=k_{d}\;\left(r_{i}-r_{i0}\right)$ based on the traction‐distance law. The parameter *k_d_* = 10 pN/µm characterizes the cell contraction strength [57, 58], **r**_*i*_ represents particle i's position vector, and **r**_*i*0_ stands for the position vector of the cell center [51, 52].

The imbalance of cell traction force during detachment of the cell rear generates the driving force for cell migration [47]. This is related to the degree of cell polarization and size. In this model, we describe the self‐driving force for active cell migration as $F_{i}^{\mathrm{driving}}=k_{dv}\;L_{max}\ln\left(AR_{i}\right)$. Here, *k_dv_* represents the intensity of the driving force of 1 pN/µm [51], while *L_max_* denotes the length of the cell's long axis, and AR reflects the cell's aspect ratio.

The bending resistance force at each node on the cell membrane can be expressed as $F_{i}^{\mathrm{bending}}=-k_{b}\;\sin\left(\theta -\theta_{0}\right)\frac{\partial \theta }{\partial s_{i}}$, where θ represents the angle between two adjacent planes, θ_0_ is the equilibrium angle, and *k_b_* denotes the bending stiffness of the cell membrane.

$F_{i}^{\mathrm{adhesion}}$ represents the force of adhesion between cells and the ECM. We identify the ECM nodes *j* within the threshold range of each cell node *i*, then $F_{i}^{\mathrm{adhesion}}=\sum_{j=1}^{N}\left(F_{max}-k_{ad}r_{ij}\right)\;$. Correspondingly, a reaction force will apply to the ECM nodes. Here, *N* represents the total number of fiber nodes within the threshold range, *F_max_* is the maximum adhesion force of each node, and *k_ad_* represents a force constant [59].

### 4.18 Statistical Analysis

All statistical analyses were performed using Origin 2025 software. When only 2 groups were involved, a standard two‐tailed t‐test was used for comparison. Comparisons of multiple groups were made using a 1‐way ANOVA analysis followed by Tukey's multiple comparison to assess intergroup differences. Results are expressed as mean ± standard deviation. Differences were considered statistically significant at P < 0.05. Differences were noted in figures as: ^****^ *p* < 0.0001, ^***^ *p* < 0.001, ^**^ *p* < 0.01, ^*^ *p* < 0.05, NS = not significant. All n numbers represent biological replicates throughout the figure captions.

## Author Contributions

**Xiangyu Xu**: methodology, software, data curation, investigation, validation, funding acquisition, visualization. **Qifeng Guan**: conceptualization, methodology, software, data curation, validation, investigation, visualization, writing – original draft, writing – review and editing. **Lizhen Wang**: formal analysis, project administration, supervision, resources, funding acquisition. **Jiakun Li**: software, validation, investigation. **Naitao Li**: methodology, software, investigation, validation, data curation. **Ping Li**: supervision, project administration, formal analysis, resources. **Kai Wang**: software, validation, investigation. **Yufan Wang**: software, validation, investigation. **Sen Hou**: conceptualization, methodology, software, data curation, supervision, formal analysis, validation, investigation, funding acquisition, writing – original draft, writing – review and editing, visualization, project administration, resources. **Jiebo Li**: writing – original draft, writing – review and editing, conceptualization, methodology, formal analysis, project administration, supervision, funding acquisition, investigation, validation, resources. **Yanzhe Fu**: methodology, validation, investigation, software, data curation, visualization. **Yubo Fan**: formal analysis, project administration, supervision, resources, funding acquisition.

## Funding

This work was supported by the National Natural Science Foundation of China 32071339 (S.H.), 92477111 (J.L.), T2288101 (L.W.), 12332019 (Y.F., Yubo Fan), U20A20390 (Y.F., Yubo Fan), 12402361 (X.X.), 12425209 (Y.F., Yubo Fan), and Fundamental Research Funds for the Central Universities (S.H.).

## Conflicts of Interest

Beihang University is patent applicants for one pending patent, application number 202510665153.6, with J.L., S.H., N.L., Q.G., Y.F. (Yanzhe Fu) and Y.F. (Yubo Fan) included among the inventors. This patent application describes three‐dimensional biological tissue fabrication using iLEAP technique. The remaining authors declare no competing interests.

## Supporting information




**Supporting File 1**: advs76938‐sup‐0001‐SuppMat.docx.

**Supporting File 2**: advs76938‐sup‐0002‐MovieS1‐S3.zip.

## Data Availability

The data that support the findings of this study are available from the corresponding author upon reasonable request.
