# Supplementary material for: Heat‐Suppressing Projection Two‐Photon Lithography Enables High‐Throughput Sub‐Micrometer Manufacturing of Biopolymer Hydrogels for Tissue Engineering
Source: Adv Sci (Weinh). 2026 Aug 24;13(63):e76938. doi: 10.1002/advs.76938 (PMC13502343; doi:10.1002/advs.76938)
Supplement: Supplementary file 1 [file ADVS-13-e76938-s001.docx]

Supporting Information

Heat-suppressing projection two-photon lithography enables high-throughput sub-micrometer manufacturing of biopolymer hydrogels for tissue engineering

Qifeng Guan, Yanzhe Fu, Xiangyu Xu, Naitao Li, Sen Hou*, Kai Wang, Yufan Wang, Jiakun Li, Ping Li, Jiebo Li*, Lizhen Wang*, Yubo Fan*

**The Stern–Volmer plots of fluorescence lifetime for the quenching process (Figure S5):**

The quenching characteristics of molecular fluorescence are typically described by the Stern-Volmer equation:

$$\begin{aligned} \frac{I_{0}}{I}=1+K_{SV}\left[ Q \right]\#\left( 1 \right) \end{aligned}$$

In the equation, *I* and *I_0_* represent the fluorescence intensities with and without the presence of quencher molecules *Q*, respectively. [*Q*] denotes the concentration of the quencher molecules, and *Ksv* is the Stern-Volmer constant. By measuring the fluorescence signal, the rate of quenching can be assessed. However, due to the complexity of accurately measuring fluorescence intensity, especially when quencher molecules exhibit absorption within the emission wavelength range of the luminescent molecules, using fluorescence intensity as a characteristic parameter is not the ideal expression for quantitatively describing the fluorescence quenching process. The Stern-Volmer equation can also be expressed in another form:

$$\begin{aligned} \frac{\tau_{0}}{\tau}=1+K_{SV}\left[ Q \right]\#\left( 2 \right) \end{aligned}$$

In the equation, *τ* represents the fluorescence lifetime in the presence of quenching, while τ0 is the fluorescence lifetime without quenching. Based on the experimental results of fluorescence lifetime quenching at varying concentrations shown in Figure 2E, the Stern-Volmer relationship is plotted in Figure S5. Using MATLAB for fitting, a *Ksv* value of 5.75 M^-1^ was obtained, and dividing by the fluorescence lifetime of 928 ps at 0 mM yields a quenching rate *kq* of 6.2 * 10^9^ M^-1^s^-1^, which is consistent with literature values. This indicates that in the presence of the quencher TEOA, EY in the singlet excited state interacts with TEOA, resulting in a shortened excited state lifetime. This process is driven by electron transfer between the TEOA and EY, and the rate constant is on a similar scale to TEOA's intersystem crossing rate, leading to competitive interaction between these two processes.

**Movie S1.** Top view of HMP array fabrication process captured by digital camera in the P-TPP system. The completion of an individual particle, demonstrated by each flash, requires less than 1s, highlighting the high efficiency of the process. Scale bar: 100 μm.

**Movie S2.** Side view of HMP array fabrication process. During the HMP fabrication process, the objective lens remains stationary while the photoresist moves along with the three-axis platform, and the photoresist at the focus position was cured. Each flash represents the completion of one HMP fabrication. Scale bar: 1 mm.

**Movie S3.** Extrusion printing of HMPs into zigzag pattern. Scale bar: 1 mm.

**Figure S1.** Performance of Irgacure 2959 and LAP in P-TPP lithography. a, The relationship between laser output power and laser intensity on the sample. b, The threshold array of hydrogel inks with LAP. Scale bar: 100 μm c, The threshold array of hydrogel inks with Irgacure 2959. Scale bar: 100 μm. d, Indirect measurement of the heat generation of various initiator-based inks (20% w/v GelMA). Temperatures reached by various inks after being exposed to laser (200 mW) for 60 s are shown in the left. An ST9450 thermal imager (SMART SENSOR) was adopted to record temperature variation. The laser spot was focused using a F=100 mm convex lens, and 20 μL ink was filled at the bottom of a 1.5 mL centrifuge tube, with the focus placed within the ink. Temperature measurements were performed during 60 s of irradiation and for 60 s after irradiation. All measurements were performed at an ambient temperature of 20 °C, and each ink sample reached thermal equilibrium with the surroundings prior to testing. LAP and I2959 were both dissolved to their maximum solubility.

**Figure S2.** The absorption spectrum of 100 mM EY with different concentrations of TEOA. The peak position and intensity of the absorption spectra remain nearly unchanged, indicating that these two components do not react in the absence of illumination.

**Figure S3.** The absorption spectrums of Irgacure 2959, LAP and EY.

**Figure S4.** The time-resolved absorption spectrums of Irgacure 2959, LAP and EY.

**Figure S5.** The Stern–Volmer plots of fluorescence lifetime for the quenching process of EY by TEOA, where τ and τ_0_ is the fluorescence lifetime in the presence and absence of TEOA.

**Figure S6.** Transient absorption spectra obtained during laser flash photolysis (with excitation at 470 nm). a, The solution containing 10 μM EY. b, The solution containing 10 μM EY and 100 mM TEOA.

**Figure S7.** Schematic diagram and photograph of the P-TPP based lithography system for rapidly producing high-precision HMPs. a, Schematic diagram. b, Photograph.

**Figure S8.** ^1^H NMR spectra of hydrogel macromers used in the formulation of printing inks. a, GelMA, b, HAA, c, Dex-GMA, d, SilMA. The degrees of substitution for modified groups were 65%, 64%, 59%, 25%, respectively.

**Figure S9.** Compression tests of different hydrogel formulations. a, Compression modulus of various hydrogels formulations after fully crosslinking (n = 4). b, Representative stress–strain curves of fully crosslinked hydrogels prepared from different printing inks: GelMA (20%, w/v), HAA (10% w/v), Dex-GMA (50% w/v), and SilMA (10% w/v).

**Figure S10.** High resolution 2D patterns. a, Fluorescence image a patterned Beihang University badge. Scale bar: 10 μm. b, Representative bright field images of printed lines with varying line widths. Scale bars: 10 μm.

**Figure S11.** Additional examples of 3D printed HMP structures. i) porous hexagonal prism, ii) cross-shaped HMP, iii-v) HMPs with identical overall porosity but varying pore numbers. Scale bars: 20 μm.

**Figure S12.** Variations in structural orientation under different writing speeds. With constant extrusion‑to‑writing speed ratio 20, a speed of 2 mm/s was selected for living cell printing due to favorable morphology and good biocompatibility. Scale bars: 100 μm.

**Figure S13.** Viability of fibroblasts co-cultured with HMSs or HMPs. a, Representative live & dead staining images after 1 and 3 days of co-culture with HMSs or HMPs. Scale bars: 100 μm. b, Quantitative analysis of the proportion of viable cells on day 1 and 3 (n = 6).

**Figure S14.** Co-culture of fibroblasts with HMPs containing multiple pores of varying diameters on day 7. a, Confocal image showing cross-sectional and longitudinal views of fibroblast infiltration into HMPs with different pore sizes (green: HMPs, blue: nucleus, red: F-actin). Scale bars: 50 μm. b, Fibroblasts infiltration proportion of different diameter pores. c, Comparison between fibroblast nuclear size and HMPs pore diameter (n = 100).

**Figure S15.** Schematic diagram and photograph of the custom-built extrusion 3D printer. a, Schematic diagram. b, Photograph.

**Figure S16.** Fibronectin coating of HMPs and co-culture with fibroblasts. a, Confocal image of fibronectin coating on the surface of a rod-shaped HMP (Green: HMP, red: FN). Scale bar: 100 μm. b, Maximum intensity projection confocal image of NIH 3T3 fibroblasts cultured within densely packed porous HMPs, demonstrating strong cell adhesion and biocompatibility (Green: HMP, blue: nucleus, red: F-actin). Scale bar: 200 μm.

**Figure S17.** Microgroove-induced alignment of fibroblasts on HMPs. a, Confocal images of used HMPs for orientation guidance and bioprinting. b, Representative images showing the orientation of NIH 3T3 fibroblasts cultured on HMPs with grooves of varying widths. Scale bars: 20 μm. c, Quantification of the 90% cell orientation distribution range on grooved HMPs (n = 25). * = p < 0.05, ** = p < 0.01, **** = p<0.0001; one-way ANOVA test.

**Figure S18.** Orientation of extrusion printed HMSs and HMPs. a, Representative confocal images of extrusion-printed HMSs and HMPs. Scale bars: 100 μm. b, Orientation distribution profiles of HMSs and HMPs following extrusion printing. c, Standard deviation of orientation angles for HMSs and HMPs, indicating the degree of alignment. **** = p<0.0001; t-test.

**Figure S19.** Extrusion 3D printing of fibroblasts-seeded HMP-based microtissues. a, Bright-field images of aligned microtissues seeded with NIH3T3 fibroblasts on day 0 and day 21 of culture. Scale bars: 400μm. b, Maximum intensity projection confocal image of fibroblast-seeded oriented microtissues on day 9, showing cell alignment along the HMPs (green: HMPs; red: F-actin). Scale bar: 500μm. c, Measured porosity of assembled HMSs and HMPs (n = 6). d, Representative tensile stress–strain curves of NIH3T3 fibroblast–seeded HMS- and HMP-based microtissues after 3 weeks of culture. NS = not significant; t-test.

**Figure S20.** Schematics of the 3D coarse-grained cell model. a, Schematic of the adhesion between cells and the matrix network. The red dots represent the boundary points on the matrix, while the black dots denote interior points. These points are interconnected via elastic springs, and the points on the cell surface can form adhesions with matrix points within a threshold distance. Discretization of dihedral angle constraints (membrane bending resistance) and cell volume constraints. b, Schematic of force components in the multicellular system: intracellular forces, intercellular forces, driving forces, and area constraint forces. c, The adhesion, contact, and attachment processes between a cell and the matrix.
